# The etiological evaluation of sensorineural hearing loss in children

**DOI:** 10.1007/s00431-019-03379-8

**Published:** 2019-05-31

**Authors:** E. A. van Beeck Calkoen, M. S. D. Engel, J. M. van de Kamp, H. G. Yntema, S.T. Goverts, M.F. Mulder, P. Merkus, E. F. Hensen

**Affiliations:** 1 Department of Otolaryngology/Head and Neck Surgery, Section Ear and Hearing, VU University Medical Center, Amsterdam, Netherlands; 2 Amsterdam Public Health research institute, Amsterdam, Netherlands; 3 Center for Diagnostics in Sensorineural Hearing Loss (CDS), VU University Medical Center, Amsterdam, Netherlands; 4 Department of Clinical Genetics, VU University Medical Center, Amsterdam, Netherlands; 5 Department of Genetics, Radboud University Medical Center, Nijmegen, Netherlands; 6 Department of Pediatrics, VU University Medical Center, Amsterdam, Netherlands; 7 Department of Otolaryngology/Head and Neck Surgery, Leiden University Medical Center, Leiden, Netherlands

**Keywords:** Bilateral hearing loss, Children, Etiology, SNHL, Unilateral hearing loss

## Abstract

This study aims to evaluate the etiology of pediatric sensorineural hearing loss (SNHL). A total of 423 children with SNHL were evaluated, with the focus on the determination of causative genetic and acquired etiologies of uni- and bilateral SNHL in relation to age at diagnosis and severity of the hearing loss. We found that a stepwise diagnostic approach comprising of imaging, genetic, and/or pediatric evaluation identified a cause for SNHL in 67% of the children. The most common causative finding in children with bilateral SNHL was causative gene variants (26%), and in children with unilateral SNHL, a structural anomaly of the temporal bone (27%). The probability of finding an etiologic diagnosis is significantly higher in children under the age of 1 year and children with profound SNHL.

*Conclusions*: With our stepwise diagnostic approach, we found a diagnostic yield of 67%. Bilateral SNHL often has a genetic cause, whereas in unilateral SNHL structural abnormalities of the labyrinth are the dominant etiologic factor. The diagnostic yield is associated with the age at detection and severity of hearing loss: the highest proportion of causative abnormalities is found in children with a young age at detection or a profound hearing loss.

**Table:** 

**What is Known:**	
*• Congenital sensorineural hearing loss is one of the most common congenital disorders*	
*• Determination of the cause is important for adequate management and prognosis and may include radiology, serology, and DNA analysis*	
**What is New:**	
*• Using a stepwise diagnostic approach, causative abnormalities are found in 67% both in uni- and bilateral SNHL, with the highest diagnostic yield in very young children and those suffering from profound hearing loss*	
*• Bilateral SNHL often has a genetic cause, whereas in unilateral SNHL structural abnormalities of the labyrinth are the dominant etiologic factor*

**Electronic supplementary material:**

The online version of this article (10.1007/s00431-019-03379-8) contains supplementary material, which is available to authorized users.

## Introduction

The prevalence of congenital sensorineural hearing loss (SNHL) is one to two per thousand live births, making it one of the most common congenital disorders [21, 25]. Early diagnosis and intervention is important in the acquisition of hearing, speech, and linguistic skills, thereby contributing to the positive development of the child [12]. Newborn hearing screening programs have been introduced, facilitating early identification of hearing-impaired children and enabling timely intervention by means of counseling, support, hearing aids, or cochlear implantation in severe cases [19, 28]. The current newborn hearing screening program in Netherlands was introduced from 2002 to 2006.

Newborn screening programs have also sparked the interest in the causes of pediatric hearing loss. Although SNHL is generally irreversible, an adequate etiological evaluation may be important for a number of reasons: prognostication of the progression of the hearing loss of the affected ear and of the unaffected ear in unilateral hearing loss, identification of associated physical conditions, identification of other family members at risk, adequate intervention if possible, and accurate counseling of the patients and their parents [2].

Imaging, DNA tests, and screening for congenital infections and metabolic diseases are frequently performed in the etiological evaluation of SNHL. It is estimated that a genetic factor is responsible for about 50% of all congenital SNHL cases, of which 70% are estimated to be non-syndromic and 30% are syndromic [17, 21, 25]. An acquired factor is found in 25% of the congenital SNHL cases [17]. These include congenital infections (TORCH: toxoplasmosis, others, rubella, cytomegalovirus, and herpes simplex viruses) and risk factors such as hypoxia during birth, hyperbilirubinemia, prematurity, and a stay at a neonatal intensive care unit (NICU) longer than 5 days. Despite etiological evaluation, the etiology of SNHL is reported to remain unknown in 25–45% of the cases [17, 21, 25].

In this study, the outcome of a stepwise diagnostic approach towards an etiological diagnosis in children with unilateral or bilateral SNHL was evaluated, with a focus on the influence of determinants such as degree and laterality of hearing loss and the age of diagnosis on the outcome of etiological diagnostics.

## Materials and methods

For the full material and methods, see Electronic Supplementary Material.

Upon parental consent, children diagnosed with bilateral or unilateral SNHL between January 2006 until January 2016 were offered etiological diagnostics by a dedicated multidisciplinary team consisting of otologists, audiologists, pediatricians, clinical geneticists, neuroradiologists and, if indicated, neurologists or ophthalmologists at the VU University Medical Center (VUmc) Amsterdam, Netherlands. Patients were referred by audiology centers, general practitioners, and otorhinolaryngologists. The majority of children were referred directly after the diagnosis of the hearing loss, but in some cases the need for etiological evaluation arises later in life, and consequently the referral takes place at an older age.

The protocols for the diagnostic evaluation of the etiology of SNHL in children are based on Dutch guidelines and the experience of the CDS team [24], and include radiology, pediatric and genetic evaluation. The outcome of radiology alone in children with SNHL has been described in more detail elsewhere [22, 23]. During the 10-year period reviewed in this study, some diagnostic protocols were altered or added to the diagnostic battery due to technological development or the evolving understanding of SNHL in children. Technical improvements have also taken place, for instance single-gene testing has been largely replaced by whole-exome sequencing (WES), and WES protocols have been improved since their introduction.

### Audiometric tests

The audiometric evaluation was performed by the referring Audiology Center or by the VUmc Audiology Center. The audiometric evaluation consisted of pure tone audiometry (PTA) if possible, auditory brainstem response using clicks (ABR), or both.

The degree of hearing loss was determined on the first available audiometric test and summarized by an average threshold at 500, 1000, 2000 and 4000 Hz on PTA or the estimated hearing threshold around 3 kHz on ABR. Children were diagnosed with bilateral SNHL if the sensorineural hearing threshold at the best hearing ear was 30 dB or more. Asymmetric bilateral SNHL was defined as 1 or more frequencies with greater than a 30 dB difference, 2 or more frequencies with greater than a 15 dB difference in threshold or 3 or more frequencies with greater than a 10 dB difference in threshold between the 2 ears (10,22). Unilateral SNHL was defined as a hearing threshold at the worst hearing ear of 30 dB or more, and a hearing threshold of 20 dB or less at the contralateral ear. Hearing loss was categorized as a slight impairment (26-40 dB), moderate impairment (41-60 dB), severe impairment (61-80 dB) and profound impairment (81 dB or greater) according to the classification of the World Health Organization (WHO) [10] . A patients’ hearing loss was graded according to the worst hearing side. In case of mixed type hearing loss, the inclusion and consecutive analyses were based on the sensorineural component only. Patients with pure conductive hearing loss were excluded from this study.

### Age

The age at detection was defined as the age at which the hearing loss was first diagnosed by the Audiology Center, either by ABR or PTA. Patients were categorized in 4 age groups: 0-1 year old, 1-6 years old, 6-12 years old and 12-18 years old.

### Evaluation etiological work-up

Patient charts were reviewed for demographic data, audiometry, and the results of dysmorphologic, pediatric, ophthalmologic, and neurologic evaluation. Furthermore, the use and results of imaging, molecular genetic testing, and laboratory tests were reviewed. Imaging consisted of computed tomography (CT) of the temporal bone and/or MR imaging of the inner ear, cerebellopontine angle, and brain. The decision to obtain imaging and the choice of the imaging modality was individualized per patient and made by the multidisciplinary team. Molecular genetic testing consisted at first of Sanger sequencing of one or several single genes (usually *GJB2*, other genes based on clinical suspicion), 201 children in our population underwent this type of genetic testing. Whole-exome-sequencing (WES) became available in a diagnostic setting in 2013. WES targeting a panel of hearing loss–related genes was performed in 204 children [29]. A congenital CMV infection was detected with polymerase chain reaction (PCR) using the dried blood spots on Guthrie cards, which are preserved for 5 years after birth in Netherlands. After this period, Guthrie cards are destroyed and reliable detection of congenital CMV is no longer possible. During the period reviewed in this study, an unrelated nationwide study into the occurrence of congenital CMV infections in children with congenital SNHL in Netherlands was taking place [10]. Some children were already evaluated for the occurrence of congenital CMV by this study before presentation for etiologic diagnosis of SNHL, in these cases, the CMV status as determined was used for the analysis in this study, and testing was not repeated at our center. Additional tests were performed when indicated by the multidisciplinary team and included metabolic screening and DNA testing for copy number variations by single-nucleotide polymorphism (SNP) array, urine screening for hematuria and proteinuria (in case of childhood onset hearing loss in boys), ECG, and evaluation of congenital infections other than CMV.

**Fig. 1:**
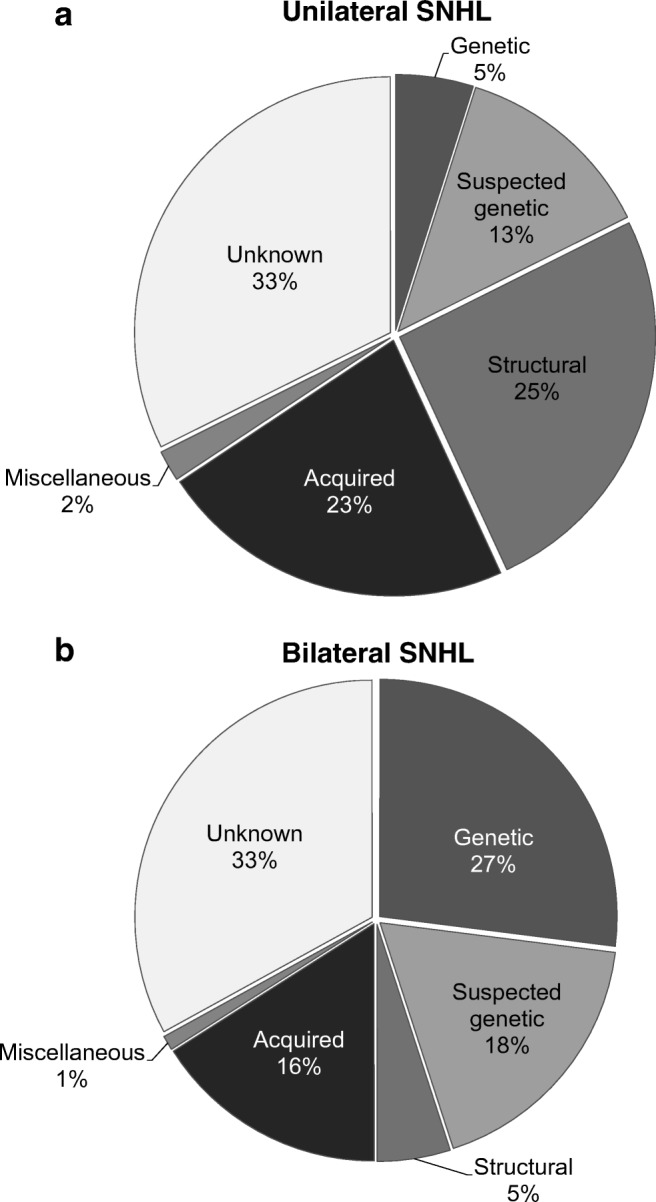
Pie charts illustrating the distribution of etiological causes of **a** unilateral and **b** bilateral sensorineural hearing loss

The etiology of SNHL was divided into different diagnostic categories: genetic, suspected genetic, structural anomalies, acquired, miscellaneous, and unknown (Fig. 1). A genetic cause was established if it was confirmed with DNA testing [29]. The SNHL was considered to be of “suspected genetic” origin if there was a strong suspicion of syndromic SNHL because of a patients’ dysmorphic features or comorbidities, a positive family history for SNHL, a gene variant of unknown pathogenicity in a gene known to be associated with hearing loss, or in some cases with a single autosomal recessive pathogenic gene variant and a specific phenotype associated with that gene. A diagnosis was categorized as “structural anomaly” if a causative abnormality was found on imaging, and no further genetic or syndromic diagnosis could be established. Acquired causes included congenital TORCH infections, meningitis, hyperbilirubinemia requiring exchange transfusion, asphyxia, neonatal intensive care stay longer than 5 days, prematurity, trauma, ototoxic drugs, and others. These risk factors were deemed causative of SNHL after exclusion of other possible causes.

## Results

### Clinical characteristics

A total of 498 children with SNHL were retrospectively evaluated. Seventy-five children were excluded from the study because further evaluation was not performed at their parent’s request, the hearing loss was not of sensorineural origin, or the sensorineural component did not exceed 30 dB. A total of 423 children were reviewed, 239 males and 184 females. Age at detection of hearing loss ranged from 1 week to 16.9 years (median age of 0.9 year). The mean age of children with bilateral SNHL was 0.7 years old, and the mean age of children with USNHL was 3.3 years old. One hundred and ninety-seven children were diagnosed with hearing loss before the age of six months (47%). They were referred directly after newborn hearing screening. Two hundred and twenty-six children (53%) were referred at an older age.

### Source of referral

Two hundred fifty-one (59%) children were referred by audiology centers, 99 (23%) by otolaryngologists, 24 (6%) by other medical specialists including pediatricians, 18 (4%) children by NICUs, 20 (5%) by child health and care service, and 11 (3%) by general practitioners.

### Hearing loss

Bilateral SNHL was diagnosed in 300 patients (71%) and unilateral SNHL in 123 children (29%). The hearing loss was most frequently profound in nature, both in uni- and bilateral SNHL (48% and 39%, respectively) (Table 1). The hearing loss was detected by automated auditory brainstem response (AABR) in 242 children (57%), at a mean age of 9 months old. The remaining children underwent pure-tone audiometry (PTA).

**Table 1:** Demographics and clinical characteristics of 423 children who underwent etiological evaluation for uni- or bilateral SNHL

Characteristics	Total	(*n*/%)	Unilateral	(*n*/%)	Bilateral	(*n*/%)
Number of patients	423	(100%)	123	(29%)	300	(71%)
Sex *n* (M/F)	M 239 F 184	(57%) (43%)	M 67 F 56	(54%) (46%)	M 172 F 128	(57%) (43%)
Hearing loss category*						
1 Slight (26–40 dB)	64	(15%)	19	(15%)	45	(15%)
2 Moderate (41–60 dB)	114	(27%)	22	(18%)	92	(31%)
3 Severe (61–80 dB) 4 Profound (80 dB or greater)	69 176	(16%) (42%)	23 59	(19%) (48%)	46 117	(15%) (39%)
Age at diagnosis (median/range) years	0.9	(0–1.9)	3.3	(0–15.8)	0.7	(0–16.9)

Age at diagnosis is the age at which the hearing loss was first diagnosed by an Audiology Center

*Hearing loss categories according to the WHO classification [11]

### Etiological work-up

**Fig. 2:**
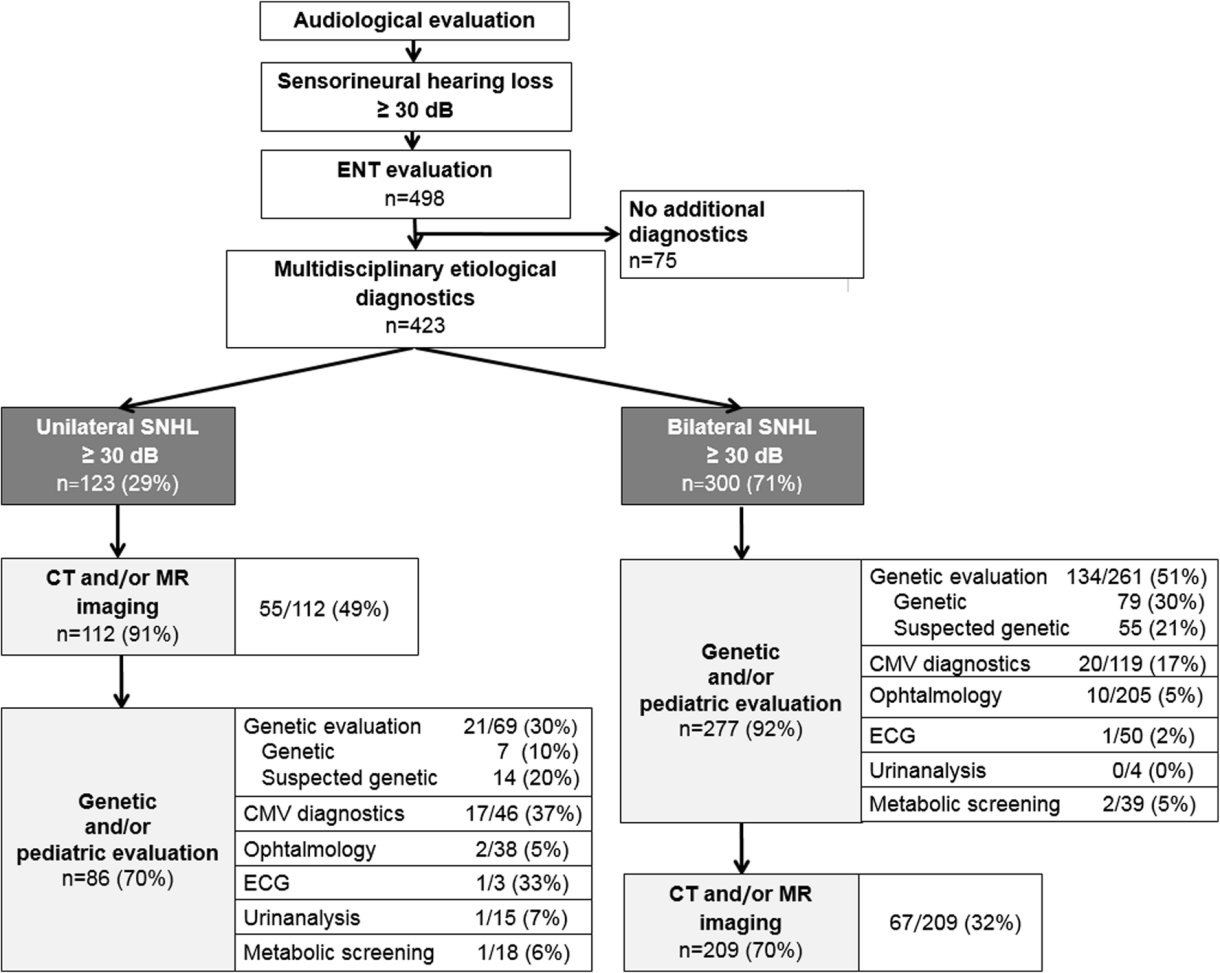
Diagnostic yield (*n*/*n* (%)) of the stepwise etiological diagnostic approach of SNHL in children. Not all children underwent all diagnostic modalities or the same diagnostic work-up: if a causative abnormality was identified in the first diagnostic step, additional diagnostics were not always deemed necessary. SNHL, sensorineural hearing loss; CT, computed tomography; MR, magnetic resonance imaging; ECG, electrocardiogram; CMV diagnostics, congenital cytomegalovirus DNA testing by PCR

The etiological evaluation was performed using a stepwise protocol in 67% of the children with USNHL and in 61% of the children with bilateral SNHL (Fig. 2). Reasons to deviate from the protocol were medical indications (including meningitis, neurodevelopment disorders, and syndromic features), a cochlear implant procedure, or parental request.

### Etiology

The etiology of the SNHL could be established in 67% of the children. The distribution of etiologic diagnoses is presented in Fig. 1. Among the children with an established etiology, the cause was most frequently genetic (*n* = 87, 31%) or acquired (*n* = 75, 26%) (Supplementary Table 3).The probability of identifying an etiologic diagnosis is significantly higher in the youngest age group (74% vs. 60%, *p* < 0.01). We also found a significantly higher diagnostic yield in the most severe hearing loss category (*p* = 0.01). As suspected, there was a significant association between age and the level of hearing loss (*p* = 0.03), i.e., the most severe hearing loss category is overrepresented in the youngest age group. Using a likelihood ratio test, the level of hearing loss was found to be of significant added value to a logistic regression model that included age only (*p* = 0.01), and vice versa (*p* = 0.04). This suggests that both severity of hearing loss and age are independent prognosticators for finding an etiologic diagnosis.

The probability of identifying a cause for SNHL did not differ between children with uni- and bilateral hearing loss (67% and 67%, respectively; *p* = 0.92). Nevertheless, the distribution of the etiologies was different for these two groups: a genetic etiology was found more frequently in the children with bilateral SNHL (27%), whereas a structural temporal bone abnormality was detected more often in children with USNHL (25%) (Table 2 A and B).

**Table 2:** A. The etiology of SNHL in children in relation to age. B. Etiology of SNHL in relation to degree of hearing loss

A.											
Etiology		Unilateral					Bilateral				
		*n* = 123					*n* = 300				
Age (years old)	Overall	0-1	1-6	6-12	12-18	Total	0-1	1-6	6-12	12-18	Total
Total	423	55 (45%)	38 (31%)	27 (22%)	3 (2%)	123	159 (53%)	86 (29%)	45 (15%)	10 (3%)	300
Genetic	87	6	-	-	1	7	50	18	8	4	80
Suspected genetic	70	8	4	2	-	14	29	14	9	4	56
Structural	48	9	10	12	2	33	8	4	3	-	15
Acquired	85	13	10	3	-	26	32	13	4	-	49
Miscellaneous	4	3	-	-	-	3	1	-	-	-	1
Unknown	139	16	14	10	-	40	39	37	21	2	99
Age: Age at which the hearing loss was first diagnosed by an Audiology Center.											
B.											
Etiology		Unilateral					Bilateral				
		*n* = 123					*n* = 300				
Degree of SNHL	Overall	26-40 dB	41-60 dB	61-80 dB	>81 dB	Total	26-40 dB	41-60 dB	61-80 dB	>81 dB	Total
Total	423	19 (15%)	22 (18%)	23 (19%)	59 (48%)	123	45 (15%)	92 (31%)	46 (15%)	117 (39%)	300
Genetic	87	1	2	-	4	7	9	28	17	26	80
Suspected genetic	70	3	1	4	6	14	10	18	13	15	56
Structural	48	4	9	2	18	33	1	4	-	10	15
Acquired	85	1	1	6	18	26	5	8	2	34	49
Miscellaneous	4	2	-	-	1	3	-	-	-	1	1
Unknown	139	8	9	11	12	40	20	34	14	31	99
Hearing loss categories according the WHO classification. SNHL: sensory neural hearing loss.											

### Genetics

A clinical evaluation by the geneticist was completed in 330/423 children. In 307 of these children, a DNA analysis was performed; 57/123 (46%) children with USNHL and 250/300 (83%) with bilateral SNHL. Reasons for genetic testing in children with USNHL included abnormalities found on CT and/or MR imaging, suspicion of syndromic SNHL, or a strong positive family history of genetic SNHL. A genetic abnormality associated with SNHL was identified in 87/307 children overall (28%). The diagnostic yield of genetic evaluation is higher in children with bilateral SNHL compared with children with USNHL (52% vs. 33% of the tested children). Genetic evaluation also revealed a significant higher proportion of genetic causes in the youngest age group (56% vs. 39%, *p* < 0.01). In contrast, the probability of finding a genetic cause was comparable between the different hearing loss categories (*p* = 0.07). Over time, the protocols for genetic evaluation have changed, from single-gene testing to WES. Especially since the introduction of WES, the diagnostic yield has increased (in this study from 26 to 36%) and will probably increase even more in the future due to improved protocols.

Of the children with a confirmed genetic cause (*n* = 87) for SNHL, 47% presented with syndromic etiology and 53% with non-syndromic etiology (Table 3; Supplementary Table 1). The most frequent genetic cause was a mutation in the GJB2 gene (27 patients, 30%), encoding Connexin 26. The most common syndromic cause was Usher syndrome (6 patients, 7%), followed by Stickler syndrome (5 patients, 6%). Ten patients (10%) were identified with Pendred syndrome.

**Table 3:** Genetic causes confirmed by DNA analysis or metabolic screening tests

Genetic	Syndrome/disease	Gene	Total (*n*)	Unilateral (*n*)	Bilateral (*n*)
			87	6	81
Non-syndromic			46	2	44
AR	DFNB1^*^	GJB2/6	27	2	25
	DFNB7/11	BSND7	1	–	1
	DFNB8	TMPRSS3	1	–	1
	DFNB9	OTOF	1	–	1
	DFNB16	STRC	5	–	5
	DFNB18	USH1C	1	–	1
	DFNB22	OTOA	2	–	2
	DFNB28	TRIOBP	1	–	1
AD	DFNA1	DIAPH1	1	–	1
	DFNA3	GJB2/6	2	–	2
	DFNA4	MYH14	1	–	1
	DFNA10	EYA4	2	–	2
	DFNA22	MYO6	1	–	1
Syndromic			41	4	37
AR	Brown-Vialetto-Van Laere syndrome	SLC52A2	1	–	1
	Chudley-McCullough syndrome	GPSM2	1	–	1
	DFNMYP syndrome^**^	SLITRK6	1	–	1
	Hurler syndrome^***^	IDUA	1	–	1
	Niemann-Pick disease type B	SMPD1	1	1	–
	Pendred syndrome	SLC26A4	10	–	10
	Usher syndrome	MYO7A, USH2A	6	–	6
	Walker-Warburg syndrome	POMT1	1	–	1
AD	Ayme-Gripp syndrome	MAF	1	–	1
	CHARGE	CHD7	3	1	2
	Primrose syndrome	ZBTB20	1	–	1
	Stickler syndrome	COL9A1	5	–	5
	Waardenburg syndrome	PAX3, SOX10	2	1	1
Chromosomal	Velo-cardio-facial syndrome	–	1	–	1
	Down syndrome	–	3	1	2
X-linked	Alport syndrome	COL4A5	1	–	1
	Hunter syndrome	IDS	1	–	1
	Turner syndrome	X(q21)	1	–	1

*AR*, autosomal recessive; *AD*, autosomal dominant; *CHARGE*, coloboma, heart defect, atresia choanae, retarded growth and development, genital and ear abnormality

^*^One patient was diagnosed with DFNB1 based on DNA confirmed DFNB1 diagnosis in a sibling with SNHL

^**^Deafness and myopia syndrome

^***^Hurler syndrome was established by metabolic screening test

Children with a suspected genetic cause (*n* = 70), presented in 43% with a positive family history for SNHL, 39% presented with a suspected syndrome associated with SNHL, and 18% had a gene mutation of unknown pathogenicity, or a single heterozygous variant in a gene (predominantly in the SLC26A4 gene) known to cause SNHL with autosomal recessive inheritance. Fourteen (20%) of these children had USNHL and 56 (80%) bilateral SNHL (Supplementary Table 2).

### Imaging

Radiologic imaging was performed in 321 children (76%), of which 112 children had USNHL and 209 had bilateral SNHL. Ninety children (28%) underwent CT as a single modality, 110 children (34%) underwent MR as a single modality, and 122 children (38%) underwent both modalities. The overall prevalence of relevant findings on imaging was 38%. Of all identified abnormalities, 60% was located within the labyrinth, 15% in the cochlear nerve, and 25% in the brain. Detailed description of the type of abnormality has been reported elsewhere [22, 23].

The diagnostic yield of imaging is higher in children with USNHL than in children with bilateral SNHL (48% vs. 32%). Profound hearing loss is associated with the highest chance of finding a radiological abnormality (*p* < 0.01). In contrast, the probability of finding an abnormality with CT or MR imaging was comparable between the different age groups (42% vs. 34%, *p* = 0.13).

### Laboratory and other tests

Congenital CMV was diagnosed in a large proportion of the tested patients (35/165; 21%). Two children had a negative CMV PCR but clear and specific clinical signs and MR findings associated with CMV infections, and were therefore diagnosed as patients with congenital CMV by the pediatric neurologist. Twenty children with a congenital CMV infection had bilateral SNHL (20/37; 54%) (Fig. 2). We identified a congenital CMV infection in a significantly higher proportion of children with USNHL compared with children with bilateral SNHL (37% vs. 17%, *p* < 0.01). In children with a congenital CMV infection, the severity of the hearing loss is usually profound, milder hearing loss was significantly less frequently observed (*p* < 0.01).

Metabolic screening tests were performed upon indication in 57/423 (13%) children. Three out of 423 (1%) patients had a metabolic disorder (mucopolysaccharidosis type I (*n* = 1), mucopolysaccharidosis type II (*n* = 1), and Niemann-Pick disease (*n* = 1). Urinalysis was performed in 19 (4%) children, one of whom had an abnormality which contributed to the diagnosis of Alport syndrome. An electrocardiogram was performed in 53 (12%) children, in two patients an abnormality was identified (in one case related to CHARGE syndrome and the other related to the disease of Niemann-Pick). Ophthalmologic examination was performed in 243 children (57%), of which 38 had USNHL and 205 bilateral SNHL. Abnormalities were identified in 84 (35%) children. Twelve children (5%) had eye abnormalities related with syndromic SNHL (i.e., retinitis pigmentosa and coloboma) or congenital CMV infection (chorioretinitis). The remaining children had refractive disorders or strabismus.

## Discussion

**Fig. 3:**
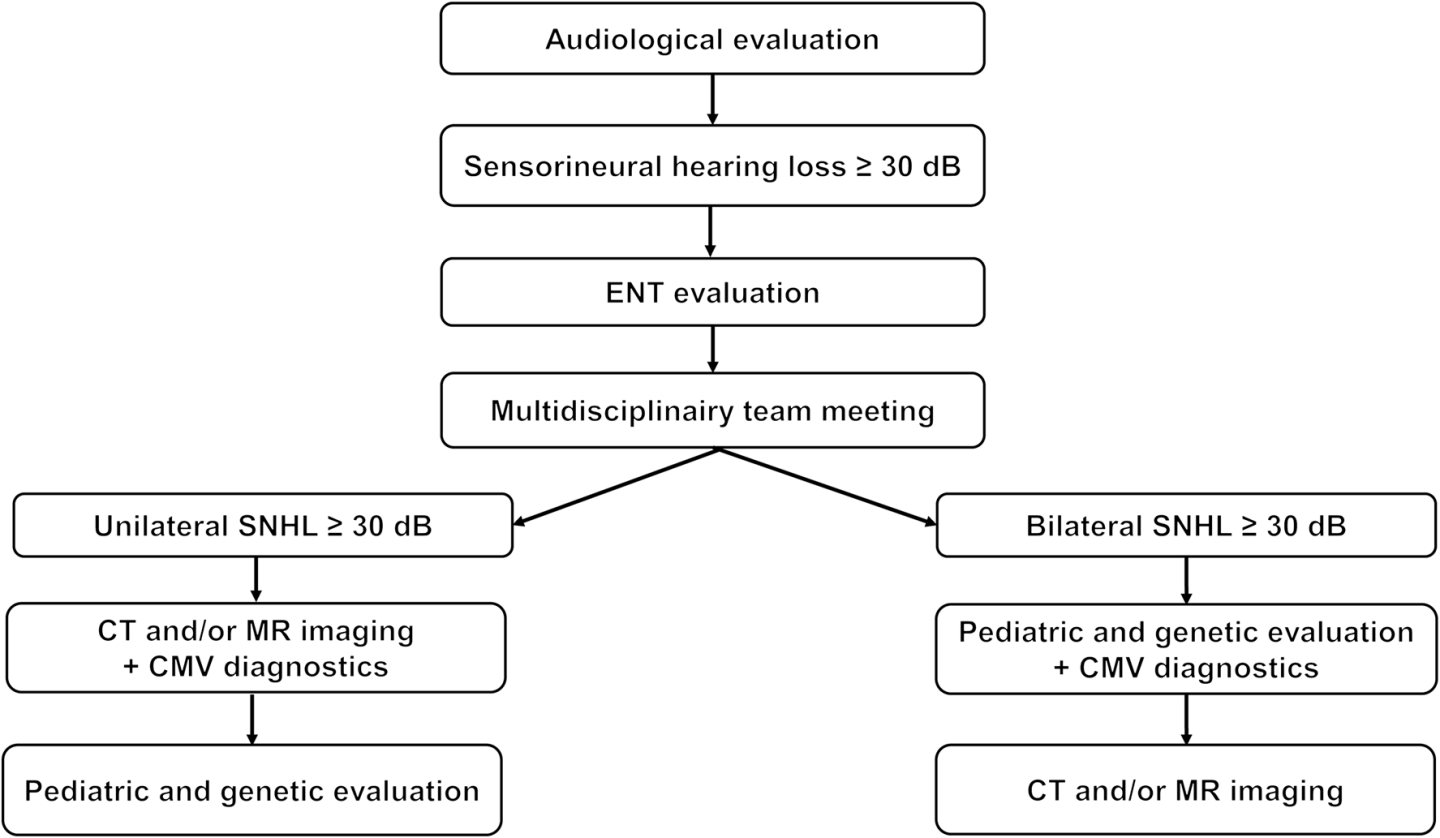
Diagnostic flow chart for children with both unilateral and bilateral sensorineural hearing loss. The results of the first step will direct further examination. Deviations from the protocol may be indicated by the multidisciplinary team (i.e., family history, medical indications, or cochlear implant procedure)

Using a stepwise diagnostic approach, we could identify an etiological diagnosis in 67% in children with uni- and bilateral SNHL (Fig. 3). This diagnostic yield is comparable with previous reports (55–81%) [3, 4, 5, 13, 18, 26].

### Unilateral vs. bilateral SNHL

The majority of children that were referred for etiological diagnostics suffered from bilateral SNHL (71%). Whereas, the chance of identifying an etiological diagnosis is comparable for USNHL and bilateral SNHL (67% vs. 67%), the distribution of etiologies differs between both groups. The most frequent etiology in children with USNHL was an isolated structural anomaly of the temporal bone, while in children with bilateral SNHL the most common cause was a genetic variant affecting gene function (in short, variant) [20, 26].

### Age and degree of hearing loss

The probability of finding an etiologic diagnosis is significantly higher in children with profound SNHL [11, 26]. In the current study, we also find a higher diagnostic yield in the youngest age group, and both age and severity of hearing loss are independent prognosticators for finding an etiologic diagnosis. However, the diagnostic yield in older pediatric patients and patients with milder hearing losses is still considerable (Table 2).

### Imaging

CT and/or MR imaging was performed in 76% of the patients. Imaging is an essential part of the etiologic analysis of USNHL because of the high prevalence of causative abnormalities that can be identified with radiology (38% in our population). In agreement with previous reports, we find a higher ratio of causative abnormalities in children with USNHL (49%) compared with children with bilateral SNHL (32%), indicating a higher diagnostic yield of imaging in children with USNHL [3, 8, 15, 27]. Because of this, we recommend to perform radiology as the first diagnostic step in patients with USNHL, and genetic evaluation as the first step in bilateral SNHL. We recommend performing CT imaging as the first modality of choice in USNHL, followed by MR imaging if CT results are negative (Supplemental Fig. 1), and MR imaging in bilateral SNHL [22, 23]. By performing the modality with the highest diagnostic yield first, additional diagnostics may be avoided, minimizing the impact on the patient and reducing costs.

### Genetic evaluation

We found that the diagnostic yield of DNA testing is considerably higher in children with bilateral SNHL than in children with USNHL (27% vs. 5%). In agreement with previous reports, variants of the *GJB2* gene were the most prevalent genetic cause (30%) [1, 8, 11, 13, 14, 16, 20, 26]. We found an equal distribution of syndromic and non-syndromic genetic etiologies, in contrast with the reported dominance of non-syndromic diagnoses [1, 18, 25]. The Pendred spectrum was the most common syndromic diagnosis in our cohort (10%). While this is in accordance with some previous reports, others find that Waardenburg or Usher syndromes are more prevalent [16, 25].

### Congenital CMV infection

Congenital CMV infection is by far the most prevalent acquired cause of congenital SNHL in this study, an observation that is in line with previous reports [6, 12]. In our study population, congenital CMV infection was found in 9% of all included children, and in 21% of children tested for CMV. With the growing recognition of congenital CMV infections as a cause of SNHL, CMV tests are nowadays performed in all children that present with SNHL, but in the first years of this study, this evaluation was not standard protocol. In addition, a neonatal screening program for congenital CMV infections has not yet been introduced in Netherlands, and the diagnosis relies on CMV PCR using the Guthrie card. As this is available until the age of 5 years in Netherlands, children that present after the age of 5 cannot be reliably tested for congenital CMV infections. The hearing loss of most of the children with congenital CMV in this cohort was classified as profound, which is in line with prior studies [7, 18]. We found that the diagnostic yield of the screening for congenital CMV infections is higher in children with USNHL than in children with bilateral SNHL (37% vs. 17%). With the possible advent of newborn screening programs for congenital CMV infections, the diagnosis of congenital CMV may become even more prevalent [7, 10].

### Limitations

Due to the stepwise approach towards the etiological diagnosis, not all children underwent the same etiological diagnostics. In addition, deviations from the protocol were sometimes indicated by the multidisciplinary team. Reasons to deviate from the protocol were medical indications (e.g., meningitis), a cochlear implant procedure or upon parental request. As a consequence, radiology was performed more often in children with unilateral SNHL, and genetic evaluation was performed more frequently in children with bilateral SNHL. The diagnostic yield of these modalities can therefore not be reliably compared between these two groups of children. If a cause was found by the first diagnostic modality, an additional diagnostic test was not always performed, and an additional cause for SNHL may have been missed in children with multiple etiologies. In our cohort, two children were identified with multiple possible causes for SNHL: in one child, a cochlear nerve aplasia was found as well as a *GJB2* gene variant, in the other, a congenital CMV infection was identified as well as Down syndrome. Performing all etiological tests in all children could possibly increase the detection rate of children suffering from multiple etiologies; however, this should be weighed against the additional costs and impact on all children undergoing etiological evaluation for SNHL.

### Conclusion and recommendations

The chance of identifying the cause of SNHL in children is high. Using our stepwise diagnostic approach, we found a diagnostic yield of 67%, both in children with uni- and bilateral SNHL (Fig. 3). Bilateral SNHL often has a genetic cause, whereas in unilateral SNHL, structural abnormalities of the labyrinth are the dominant etiologic factor. Based on these results we start the etiologic diagnostic work-up with genetic evaluation in children with bilateral SNHL, and with radiology in children with USNHL (Supplementary Fig. 1). Congenital CMV infections are a cause for both uni- and bilateral SNHL, and we recommend evaluation of congenital CMV infections in all children that present with SNHL. The highest proportion of causative abnormalities is found in children younger than 1 year and in children suffering from profound hearing loss. However, the diagnostic yield in older pediatric patients and patients with milder hearing losses is still considerable. We therefore offer etiological diagnostics to all pediatric patients with SNHL exceeding 30 dB, irrespective of age or degree of hearing loss.

## Electronic supplementary material

Supplementary figure 1 Flowchart of imaging in children with USNHL. USNHL = unilateral sensorineural hearing loss. CT = computed tomography. MR = magnetic resonance imaging. IAC = internal auditory canal. (PDF 33 kb)

ESM 1 (DOCX 20 kb)

## Abbreviation

AABR: Automated auditory brainstem response
AC: Audiology center
AD: Autosomal dominant
AR: Autosomal recessive
CDS: Center of diagnostics of SNHL
CHARGE: Coloboma, heart defect, atresia choanae, retarded growth and development, genital and ear abnormality
CMV: Cytomegalovirus
CT: Computed tomography
EVA: Enlarged vestibular aqueduct
IAC: Internal auditory canal
MR: Magnetic resonance imaging
NGS: Next-generation sequencing
PCR: Polymerase chain reaction
PTA: Pure-tone audiometry
SNHL: Sensory neural hearing loss
TORCH: Toxoplasmosis, other, rubella, cytomegalovirus (CMV), and herpes infections
USNHL: Unilateral sensory neural hearing loss
VUmc: VU University Medical Center
WES: Whole-exome sequencing

## References

[1] Alford RL, Arnos KS, Fox M, Lin JW, Palmer CG, Rehm HL (2014). American College of Medical Genetics and Genomics guideline for the clinical evaluation and etiologic diagnosis of hearing loss. Genet Med.

[2] American Academy of Pediatrics, Joint Committee on Infant Hearing (2007) Year 2007 position statement: principles and guidelines for early hearing detection and intervention programs. Pediatrics 120(4):898–921 10.1542/peds.2007-2333 17908777

[3] Bamiou DE, Phelps P, Sirimanna T (2000). Temporal bone computed tomography findings in bilateral sensorineural hearing loss. Arch Dis Child.

[4] Declau F, Boudewyns A, van den Ende J, Peeters A, van den Heyning P (2008). Etiologic and audiologic evaluations after universal neonatal hearing screening: analysis of 170 referred neonates. Pediatrics.

[5] Deklerck AN, Acke FR, Janssens S, de Leenheer EM (2015). Etiological approach in patients with unidentified hearing loss. Int J Pediatr Otorhinolaryngol.

[6] Fowler KB, Boppana SB (2006). Congenital cytomegalo (CMV) infection and hearing deficit. J Clin Virol.

[7] Grosse SD, Ross DS, Dollard SC (2008). Congenital cytomegalovirus (CMV) infection as a cause of permanent bilateral hearing loss: a quantitative assessment. J Clin Virol.

[8] Haffey T, Fowler N, Anne S (2013). Evaluation of unilateral sensorineural hearing loss in the pediatric patient. Int J Pediatr Otorhinolaryngol.

[9] Harvest S (2012) Community based rehabilition: promoting ear and hearing care through CBR. World Health Organization, India, p 8

[10] Korver AM, de Vries JJ, Konings S, de Jong JW, Dekker FW, Vossen AC, Frijns JH, Oudesluys-murphy AM, DECIBEL collaborative study group (2009) DECIBEL study: congenital cytomegalovirus infection in young children with permanent bilateral hearing impairment in the Netherlands. J Clin Virol 46(Suppl 4):S27–31 10.1016/j.jcv.2009.09.007 19836301

[11] Korver A, Admiraal RJC, Kant GK, Dekker FW, Wever CC, Kunst HPM, Frijns JH (2011). Causes of permanent childhood hearing impairment. Laryngoscope.

[12] Kral A, O’Donoghue M (2010). Profound deafness in childhood. N Engl J Med.

[13] Lammens F, Verhaert N, Devriendt K, Debruyne F, Desloovere C (2013). Aetiology of congenital hearing loss: a cohort review of 569 subjects. Int J Pediatr Otorhinolaryngol.

[14] Lin JW, Chowdhury N, Mody A, Tonini R, Emery C, Haymond J, Oghalai JS (2011). Comprehensive battery for evaluating SNHL in children. Int J Pediatr Otorhinolaryngol.

[15] McClay JE, Booth TN, Parry DA, Johnson R, Roland P (2008). Evaluation of pediatric sensorineural hearing loss with magnetic resonance imaging. Arch Otolaryngol Head Neck Surg.

[16] Mehta D, Noon S, Schwartz E,Wilken A, Bedoukia EC, Scarana I, Crenshaw BE, Krantz ID (2016) Outcomes of evaluation and testing of 660 individuals with hearing loss in a pediatric genetics of hearing loss clinic. Am J Med Genet A 170(10):2523-2530 10.1002/ajmg.a.37855 27480936

[17] Morton C, Nance W (2006). Newborn hearing screening – a silent revolution. N Engl J Med.

[18] Morzaria S, Westerberg BD, Kozak FK (2004) Systematic review of the etiology of bilateral sensorineural hearing loss in children. Int J Pediatr Otorhinolaryngol 68(9):1193–1198 10.1016/j.ijporl.2004.04.013 15302152

[19] Pimperton H, Kennedy C (2012). The impact of early identification of permanent childhood hearing impairment on speech and language outcomes. Arch Dis Child.

[20] Preciado DA, Lawson L, Madden C, Myer D, Ngo C, Bradshaw J, Choo DI, Greinwald JH (2005). Improved diagnostic effectiveness with a sequential diagnostic paradigm in idiopathic pediatric sensorineural hearing loss. Otology & Neurotology.

[21] Smith RJH, Bale JF, White KR (2005). SNHL in children. Lancet.

[22] van Beeck Calkoen EA, Sanchez Aliaga E, Merkus P, Smit CF, van de Kamp JM, Mulder MF, Goverts ST, Hensen EF (2017). High prevalence of abnormalities on CT and MR imaging in children with unilateral sensorineural hearing loss irrespective of age or degree of hearing loss. Int J Pediatr Otorhinolaryngol.

[23] van Beeck Calkoen EA, Merkus P, Goverts ST, van de Kamp JM, Mulder MF, Sanchez Aliaga E, Hensen EF (2018). Evaluation of the outcome of CT and MR imaging in pediatric patients with bilateral sensorineural hearing loss. Int J Pediatr Otorhinolaryngol.

[24] Vereniging klinische genetica Nederland (2012) Richtlijn etiologisch onderzoek naar slechthorendheid op de kinderleeftijd

[25] White KR (2004). Early hearing detection and intervention programs: opportunities for genetic services. Am J Med Genet A.

[26] Wiley S, Arjmand E, Meinzen-Derr J, Dixon M (2011). Findings from multidisciplinary evaluation of children with permanent hearing loss. Int J Pediatr Ororhinolaryngol.

[27] Wormald R, Viani L, Lynch SA, Green AJ (2010). Sensorineural hearing loss in children. Ir Med J.

[28] Yoshinago-Itano C (2003). From screening to early identifcation and intervention: discovering predictors to successful outcomes for children with significant hearing loss. J Deaf Stud Deaf Educ.

[29] Zazo Seco C, Wesorp M, Feenstra I, Pfundt R, Hehir-Kwa JY, Lelieveld SH, Castelein S, Gilissen C, de Wijs IJ, Admiraal RJ, Pennings RJ, Kunst HP, van de Kamp JM, Tamminga S, Houweling AC, Plomp AS, Maas SM, de Koning Gans PA, Kant SG, de Geus CM, Frints SG, Vanhoutte EK, van Dooren MF, van den Boogaard MH, Scheffer H, Nelen M, Kremer H, Hoefsloot L, Scharders M, Yntema HG (2017). The diagnostic yield of whole-exome sequencing targeting a gene panel for hearing impairment in the Netherlands. Eur J Hum Genet.

