# Supplementary figures and images for: The etiological evaluation of sensorineural hearing loss in children
Source: Eur J Pediatr. 2019 May 31;178(8):1195–205. doi: 10.1007/s00431-019-03379-8 (PMC6647487; doi:10.1007/s00431-019-03379-8)

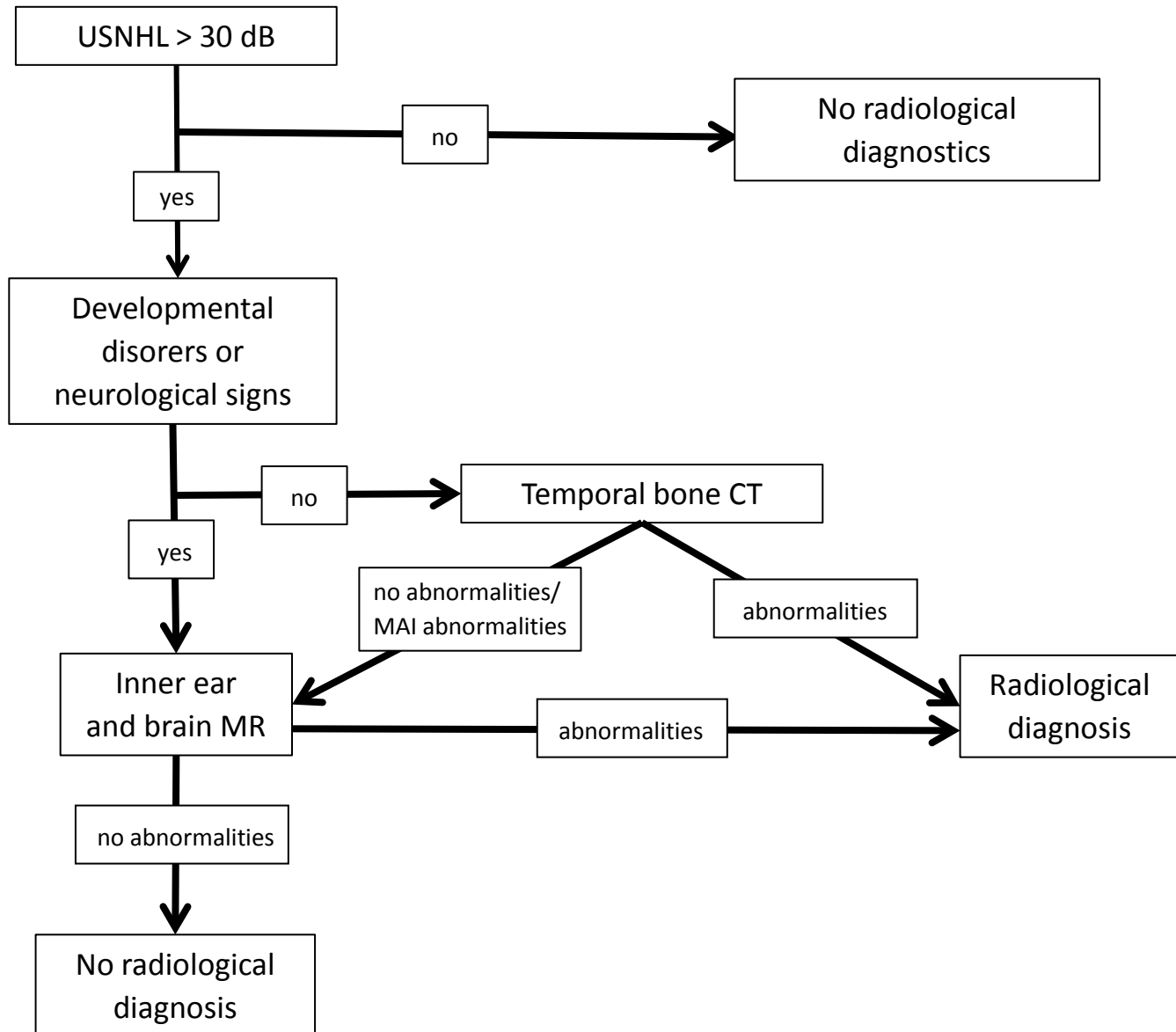

Supplement: Supplementary file 1 — Flowchart of imaging in children with USNHL. USNHL = unilateral sensorineural hearing loss. CT = computed tomography. MR = magnetic resonance imaging. IAC = internal auditory canal. (PDF 33 kb) [file 431_2019_3379_MOESM1_ESM.pdf]
