# Supplementary material for: The etiological evaluation of sensorineural hearing loss in children
Source: Eur J Pediatr. 2019 May 31;178(8):1195–205. doi: 10.1007/s00431-019-03379-8 (PMC6647487; doi:10.1007/s00431-019-03379-8)
Supplement: Supplementary file 2 — (DOCX 20 kb) [file 431_2019_3379_MOESM2_ESM.docx]

**Supplementary table 1** *List of gene variants*

| **Gene** | **Variant 1 nucleotide change** | **Variant 2 nucleotide change** | **Zygosity** | **Segragation with HI** |
| --- | --- | --- | --- | --- |
| USH2A | C.7950dup; p.(Asn2651fs) | C.10561T>C; p.(Trp3521Arg) | compound het | Yes |
| USH2A | c.1256G>T; p.(Cys419Phe) | c.2299del; p.(Glu767fs) | compound het | Yes |
| USH2A | c. 15089C>A;p.(Ser5030X) | (c.6713A>C;p.Glu2238Ala) + (c. 12294+3A>G;p.?) | compound het | Yes |
| MYO6 | c.-3_1delinsTT (p.?) |  | het | Yes |
| GJB2 | c.109G>A; p.(Val37Il) | c.109G>A; p.(Val37Il) | hom | ND |
| GJB2 | c.109G>A; p.(Val37Il) | c.109G>A; p.(Val37Il) | hom | Yes |
| GJB2 | c.35del; p.(Glyfs) | c.35del; p.(Glyfs) | hom | ND |
| GJB2/6 | c.35del; p.(Gly12fs) | c.35 del; p.(Gly12fs) | hom | ND |
| GJB2 | c.35del; p.(Gly12fs) | c.35del; p.(Gly12fs) | hom | Yes |
| GJB2 | c.35del; p.(Gly12fs) | c.35del; p.(Gly12fs) | hom | Yes |
| GJB2 | c.35del; p.(Gly12fs) | c.35del; p.(Gly12fs) | hom | ND |
| SOX10 | c.482G>A; p.(Arg161His) |  | het | De novo |
| PAX3 | c.808C>T; p.(Arg270Cys) |  | het | Yes |
| CHD7 | c.8077-2A>C (spl.?) |  | het | ND |
| CHD7 | c.2520G>A; p.(Trp840*) |  | het | De novo |
| GJB2 | c.223C>T; p.(Arg75Trp) |  | het | Yes |
| GPSM2 | c.742del; p.(Gly249fs) | c.742del; p.(Gly249fs) | hom | Yes |
| EYA4 | c.1234del; p.(Met412X) |  | het | ND |
| GJB2 | c.126G>T; p.(Glu42Asp) |  | het | Yes |
| GJB2 | c.109G>A; p.(Val37Ile) | c.109G>A; p.(Val37Ile) | hom | ND |
| SMPD1 | c.308T>C; p.(Leu103Pro) | c.308T>C; p.(Leu103Pro) | hom | Yes |
| COL4A5 | c.2513dupT; p.(Leu838Phefs*17) |  | het | ND |
| USH2A | c.7121-8313_11048-962delins12 (deletion exon 38 - 56) | c.5813G>A; p.(Gly1938Asp) + c.15017C>T; p.(Thr5006Met) | compound het | Yes |
| GJB2 | (c.109G>A(p.Val37Ile) | (c.109G>A(p.Val37Ile) | hom | Yes |
| GJB2/6 | c.35 delG; p.(Gly12fs) | del 342kb | compound het | Yes |
| GJB2 | c.250G>A; p.(Val84Met) | p.109G>A; p.(Val37Ile) | compound het | Yes |
| MYO7A | c.3109-2A>G (r.spl?) | c.3476G>T; (p.(Gly1159Val) | compound het | Yes |
| ZBTB20 | c.1794C>G; p.(Phe598Leu) |  | het | De novo |
| G JB 2 | c.35del; p.(Gly12fs) | c.71G>A; p.(Trp24X) | compound het | Yes |
| DIAPH1 | c.3637C>T; p.(Arg1213X) |  | het | ND |
| GJB2/6 | c.269T>C; p.(Leu90Pro) |  | compound het | Yes |
| SLC26A4 | c.1790T>C; p .(Leu597Ser) | c.1790T>C; p .(Leu597Ser) | hom | Yes |
| IDS | c,998C>T; p.(Ser33Leu) |  | hemi | Yes |
| COL9A2 | c.406C>T; p.(Arg136) | c.406C>T; p.(Arg136) | hom | ND |
| GJB2 | c.35del; p(Gly12fs) | c.358_360del; p.(Glu120del) | compound het | Yes |
| SCL26A4 | c.412G>T; p.(Val138Phe) | c.707T>C; p.(Leu236Pro) | het | Yes |
| TRIOBP | c.3460_3461; p.(Leu1154fs) | c.3232dup; p.(ARg1078fs) | compound het | Yes |
| SLC26A4 | c.505del; p.(Thr169fs) | c. 1334T>G; p.(Leu445Trp) | compound het | Yes |
| CHD7 | c,2959C>T; p.(arg987X) |  | het | ND |
| MAF | c.197C>G; p.(Ser66Trp) |  | het | De novo |
| GJB2 | c.-3170G>A | c.-3170G>A | hom | ND |
| OTOF | c.505C>T; p.(Arg169Trp) | c.505C>T; p.(Arg169Trp) | hom | Yes |
| GJB2 | c.-23+1G>A | c.71G>A; p.(Trp24X) | compound het | Yes |
| GJB2 | c.358_360 del; p.(Glu120del) | c.-23+1G>A | compound het | ND |
| TMPRSS3 | c.916G>T; p.(Ala306Thr) | c.413C>A; p.(Ala138Gln) | compound het | Yes |
| COL11A2 | c.3877C>T; p.(Arg1293*) |  | het | De novo |
| GJB2 | c.35del; p.(Gly12fs) | c.101T>C; p.(Met34Thr) | compound het | Yes |
| CATSPER2 and STRC | 15q15.3 | 15q15.3 | hom | ND |
| CATSPER2 and STRC | 15q15.3 | 15q15.3 | hom | ND |
| GJB2 | c.35del; p.(Gly12fs) | c.109G>A; p.(Val37Ile) | compound het | Yes |
| MYO7A | c.5618G>A; p.(1873Gln) | c.6028G>A; p.(Asp2010Asn) | compound het | Yes |
| SLC26A4 | c.707T>C; p.(Leu236Pro) | c.1334T>G; p.(Leu445Trp) | compound het | Yes |
| OTOG | c.1009C>T; p.(Gln337*) | c.7454del; p.(Arg2485fs) | compound het | ND |
| SLC52A2 | c.167C>T; p.(Ala56Val) | c.593G>A; p.(Trp198*) | compound het | ND |
| GJB2 | c.35del; p.(Giy12fs) | c.35del; p.(Giy12fs) | hom | Yes |
| CATSPER2 and STRC | 15q15.3 | 15q15.3 | hom | ND |
| OTOA | c.2207G>A; p.(Gly736Glu) | exon 1 - 21 | compound het | Yes |
| STRC | exon 19-26 | exon 19-26 | hom | Yes |
| SLITRK6 | c.1438del; p.(Ser480fs) | c.1438del; p.(Ser480fs) | hom | Yes |
| COL9A2 | c.406C>T; p. (Arg136*) | c.406C>T; p. (Arg136*) | hom | Yes |
| SLC26A4 | c.85G>C; p.(Glu29Gln) | c.1151A>G; p.(Glu384Gly) | compound het | Yes |
| GJB2 | c.235del; p.(Leu79fs) | c.427C>T;p. (Arg143Trp) | compound het | ND |
| GJB2 | (c.35del p.(Gly12fs) | c.427C>T: p.(arg143Trp) | compound het | ND |
| COL11A1 | c.1798C>T; p.(Arg600X) |  | het | De novo |
| GJB2 | c.313_326del; p.(Lys105fs) | c.101T>C; p.(Met34Thr) | compound het | Yes |
| TMC1 | c.247G>T; p.(Glu83X) | c.1763+3A>G; (r.spl?) | compound het | Yes |
| OTOA | c.2359G>T; p.(Gly787X) | c.2359G>T; p.(Gly787X) | hom | ND |
| STRC | exon 19 - 26 | exon 19 - 26 | hom | Yes |
| GJB2 | c.35del; p.(Gly12fs) | c.-23+1G>A (r.spl?) | compound het | Yes |
| GJB2 | c.109G>A; p.(Val37Ile) | c.44A>C; p.(Lys15Thr) | compound het | Yes |
| SLC26A4 | c.707T>C (p.Leu236Pro) | c.1342-2A>C | compound het | Yes |
| GJB2 | c.35del; p.(Gly12fs) | c.269T>C; p.(Leu90Pro) | compound het | Yes |
| SLC26A4 | c.1001+1G>A | no second variant found | het | ND |
| SLC26A4 | c.1784G>A; p.(Gly595Glu) | no second variant found | het | ND |
| SLC26A4 | c.1001+1G>A (p.?) | no second variant found | het | ND |
| SLC26A4 | c.84C>A; p.(Ser28Arg) | no second variant found | het | Yes |
| SLC26A4 | c.412G>T; p.(Val138Phe) | no second variant found | het | ND |
| SLC26A4 | c.1246A>C; p.(Thr416Pro) | no second variant found | het | ND |
| SLC26A4 | c.707T>C; p.(Leu236Pro) | no second variant found | het | Yes |
| SLC26A4 | c.1198del; p.(Cys400fs) | no second variant found | het | ND |
| CHD23 | c.5117G>A; p.(Arg1706His) | c.5945A>G; p.(Asn1982Ser) | compound het | Yes |
| LOXHD1 | c.2696G>C; p.(Arg899Pro) | c.5934C>T; (r.spl?) | compound het | Yes |

Abbreviaties: Het: hetezygous. Hom: homozygous. Hemi: hemizygous. ND: not determinded or not conclusive.

**Supplementary table 2** *Overview of the suspected genetic causes.*

| **Suspected genetic category** | **N= 70** |
| --- | --- |
| Non syndromal  Positive family history for SNHL | 35  29 |
| Syndromal | 27 |
| Single heterozygote SLC26A4 mutation  Unilateral EVA  Bilateral EVA | 8  1  7 |

**Supplementary table 3** *Overview of the acquired causes and risk factors for SNHL identified in this study, categorized according to the American Academy of Pediatrics.*

| **Etiology: acquired causes and risk factors** | **Number** |
| --- | --- |
| Congenital TORCH infection  CMV | 38  36 |
| Meningitis | 13 |
| Hyperbilirubinemia requiring exchange transfusion | 2 |
| Asphyxia | 8 |
| Pre- and dysmaturity, NICU stay longer than 5 days | 14 |
| Trauma | 2 |
| Total | 75 |
